# The Effects of Dark Incubation on Cellular Metabolism of the Wild Type Cyanobacterium *Synechocystis* sp. PCC 6803 and a Mutant Lacking the Transcriptional Regulator cyAbrB2

**DOI:** 10.3390/life4040770

**Published:** 2014-11-21

**Authors:** Masamitsu Hanai, Yusuke Sato, Atsuko Miyagi, Maki Kawai-Yamada, Kyoko Tanaka, Yasuko Kaneko, Yoshitaka Nishiyama, Yukako Hihara

**Affiliations:** 1 Graduate School of Science and Engineering, Saitama University, Saitama 338-8570, Japan; E-Mails: s13mb111@mail.saitama-u.ac.jp (M.H.); s14mb115@mail.saitama-u.ac.jp (Y.S.); miyagi@mail.saitama-u.ac.jp (A.M.); mkawai@mail.saitama-u.ac.jp (M.K.-Y.); kytanaka@mail.saitama-u.ac.jp (K.T.); yakaneko@mail.saitama-u.ac.jp (Y.K.); nishiyama@molbiol.saitama-u.ac.jp (Y.N.); 2 Institute for Environmental Science and Technology, Saitama University, Saitama 338-8570, Japan; 3 Precursory Research for Embryonic Science and Technology (PRESTO), Japan Science and Technology Agency (JST), Saitama 332-0012, Japan; 4 Core Research of Evolutional Science and Technology (CREST), Japan Science and Technology Agency (JST), Saitama 332-0012, Japan

**Keywords:** capillary electrophoresis-mass spectrometry, cyAbrB2, dark, glycogen, metabolic regulation, *Synechocystis*

## Abstract

The cyAbrB2 transcriptional regulator is essential for active sugar catabolism in *Synechocystis* sp. PCC 6803 grown under light conditions. In the light-grown *cyabrB2*-disrupted mutant, glycogen granules and sugar phosphates corresponding to early steps in the glycolytic pathway accumulated to higher levels than those in the wild-type (WT) strain, whereas the amounts of 3-phosphoglycerate, phosphoenolpyruvate and ribulose 1,5-bisphosphate were significantly lower. We further determined that accumulated glycogen granules in the mutant could be actively catabolized under dark conditions. Differences in metabolite levels between WT and the mutant became less substantial during dark incubation due to a general quantitative decrease in metabolite levels. Notable exceptions, however, were increases in 2-oxoglutarate, histidine, ornithine and citrulline in the WT but not in the mutant. The amounts of cyAbrBs were highly responsive to the availability of light both in transcript and protein levels. When grown under light-dark cycle conditions, diurnal oscillatory pattern of glycogen content of the mutant was lost after the second dark period. These observations indicate that cyAbrB2 is dispensable for activation of sugar catabolism under dark conditions but involved in the proper switching between day and night metabolisms.

## 1. Introduction

Cyanobacteria are highly versatile organisms that modulate their metabolic activity in response to the availability of light and carbon [1,2,3]. In the presence of light they grow photoautotrophically, performing oxygenic photosynthesis, and the ATP and NADPH generated by photosynthetic electron transport are used in cellular metabolic processes, such as CO_2_ fixation via the Calvin cycle and nitrogen assimilation via the glutamine synthetase-glutamate synthase (GS-GOGAT) pathway. The fixed carbon provides carbon skeletons for various metabolic reactions, or is stored in the form of glycogen. When cells are placed in the dark, CO_2_ fixation ceases and glycogen break down occurs. The resulting sugars are catabolized via the oxidative pentose phosphate (OPP) pathway, the glycolytic pathway and the tricarboxylic acid (TCA) cycle to produce ATP, NAD(P)H and carbon compounds required for survival during the dark period. Certain cyanobacterial lineages can take up and catabolize exogenous sugar compounds to grow heterotrophically. Since cyanobacteria have no organelles, most of abovementioned CO_2_ fixation, sugar catabolic and anabolic pathways are located in cytoplasm and several enzymes are shared among these metabolic reactions [4]. Thus, coordination of these metabolic pathways is a prerequisite for optimal cyanobacterial growth under changing nutrient conditions.

Recently, cyanobacteria-specific transcriptional regulators of the AbrB superfamily, termed cyAbrB proteins, were shown to be involved in metabolic regulation. They are structurally distinct from AbrB-type regulators in other bacterial species in that their DNA-binding domain is located in the C-terminal region rather the N-terminus [5]. All known cyanobacterial genomes possess multiple copies of genes encoding cyAbrB transcriptional regulators. For example, the genome of the unicellular cyanobacterium *Synechocystis* sp. PCC 6803, has two cyAbrB genes, *cyabrB1* (sll0359) and *cyabrB2* (sll0822), as well as several *abrB* genes encoding AbrB-type regulators with an N-terminal DNA-binding domain.

We previously reported that *cyabrB1*, but not *cyabrB2*, is an essential gene in the glucose-tolerant wild-type (WT) strain (GT strain) of *Synechocystis* sp. PCC 6803 [5] and several studies of a *cyabrB2*-disrupted mutant (∆*cyabrB2*) have revealed that cyAbrB2 plays a key role in regulating diverse aspects of cellular metabolism. We observed that cyAbrB2 positively regulates nitrogen-regulated genes, such as *urtA*, *amt1*, *glnB*, *sigE* and the *nrt* operon, under ambient CO_2_ conditions [5]. Additionally, Lieman-Hurwitz *et al.* [6] demonstrated that cyAbrB2 functions as a repressor of genes involved in inorganic carbon (Ci) uptake under high (5%) CO_2_ conditions, and they observed that ∆*cyabrB2* exhibits a much higher photosynthetic affinity for Ci than WT cells under high CO_2_ conditions. Dutheil *et al.* [7] reported that cyAbrB2 in the original glucose-sensitive WT *Synechocystis* strain binds to the upstream region of the *hox* operon encoding the bidirectional Ni-Fe hydrogenase, where it functions as a repressor. Furthermore, the same group found through a transcriptome analysis that cyAbrB2 regulates a large number of chromosomal genes related to transcriptional regulation, metal transport and protection against oxidative stress [8].

Microscopic observation revealed the accumulation of substantial numbers of glycogen granules in the spaces between the thylakoid membranes of the ∆*cyabrB2* mutant but not in the WT (GT strain) under photoautotrophic conditions, suggesting the existence of metabolic defects in the mutant [9]. Subsequent metabolome analysis further established that the levels of sugar phosphates were higher in ∆*cyabrB2* than in WT under photoautotrophic conditions, whereas those of several downstream metabolites, such as pyruvate and 2-oxoglutarate (2OG), were lower [10]. Incubation of the mutant cells under photomixotrophic conditions with glucose in the presence of light resulted in more severe phenotypes, with a dense accumulation of glycogen granules throughout the cell and a major decline in CO_2_ fixation, such that the mutant cells did not survive after 24 h [10]. Thus, the partitioning of carbon between storage forms and soluble forms used for biosynthetic purposes appeared to be defective in the mutant. This disorder may cause inactivation of cellular metabolism and subsequent loss of viability under photomixotrophic conditions.

Excess carbon accumulating in the form of glycogen granules in the ∆*cyabrB2* mutant represents a potentially valuable source of materials for the production of biofuels and other industrial products if that carbon could be redirected through a desired biosynthetic pathway by genetic engineering. We have tried such metabolic engineering by disrupting the *agp* gene (slr1176) encoding ADP-glucose pyrophosphorylase (AGPase). AGPase catalyzes the synthesis of ADP-glucose and inorganic pyrophosphate from ATP and glucose-1-phophate. Since ADP-glucose is the substrate for glycogen synthesis and *agp* is a single copy gene, a complete blockage of glycogen biosynthesis can be achieved by deletion of the *agp* gene, as has been demonstrated in the WT strains of *Synechocystis* sp. PCC 6803 [11] and *Synechococcus* sp. PCC 7942 [12]. However, when we disrupted the *agp* gene in the ∆*cyabrB2* background, only partially-segregated mutant line was obtained and cellular glycogen content did not decrease (data not shown). We conclude from these results that accumulation of glycogen granules is essential for the survival of the ∆*cyabrB2* mutant.

Here, we report that glycogen metabolism is substantially different in the dark-incubated ∆*cyabrB2* mutant cells. The rate of decrease in cellular glycogen content in the mutant was similar to, or somewhat higher than, that in WT during dark incubation, raising questions concerning the role of cyAbrB2 in metabolic regulation under dark conditions. To elucidate the function of cyAbrB2 on cellular metabolism in the dark we performed a metabolome analysis of dark-incubated WT and the ∆*cyabrB2* cells and examined their growth and fluctuations in glycogen content under diurnal light-dark cycles.

## 2. Experimental Section

### 2.1. Strains and Culture Conditions

A glucose-tolerant strain (GT strain) of *Synechocystis* sp. PCC 6803 was grown at 32 °C in BG-11 medium containing 20 mM HEPES-NaOH, pH 7.0, under continuous illumination at 20 μmol photons m**^−^**^2^ s**^−^**^1^ or a 12 h:12 h light-dark cycle, with air bubbling through the culture. The ∆*cyabrB2*-disrupted mutant [5] was grown under the same conditions, except that 20 μg mL**^−^**^1^ kanamycin was added to the medium. Cell density was estimated by measuring optical density at 730 nm (OD_730_) using a spectrophotometer (model UV-160A; Shimadzu, Kyoto, Japan).

### 2.2. Determination of Glycogen Content

Glycogen content was determined as described in Suzuki *et al.* [12]. Briefly, the cell pellet was resuspended in absolute methanol and kept at −20 °C overnight. After centrifugation, the dried pellet was resuspended and incubated at 100 °C for 40 min, and then at 40 °C for 1 h with glucoamylase. Glycogen content was determined enzymatically by addition of hexokinase and glucose 6-phosphate dehydrogenase. The amount of glucose moieties derived from glycogen was determined as the increase in OD_340_. Cellular glycogen content was calculated in terms of μg of glucose mL**^−^**^1^·OD_730_**^−^**^1^ using the extinction coefficient of NADPH at 340 nm (6.22 × 10^3^ M**^−^**^1^ cm**^−^**^1^).

### 2.3. Electron Microscopy

Electron microscopy was performed as described previously [9].

### 2.4. Measurement of Respiratory Activity

Cultures grown to an OD_730_ of 0.5 were concentrated approximately five-fold by centrifugation and a 1 mL aliquot was placed in a Clark-type oxygen electrode chamber and stirred gently at 30 °C. Oxygen consumption rate was measured in the dark and calculated in terms of μmol of oxygen consumed OD_730_**^−^**^1^·mL**^−^**^1^·h**^−^**^1^ and μmol of oxygen consumed 10^9^ cells**^−^**^1^·h**^−^**^1^.

### 2.5. Metabolome Analysis

The metabolome analysis was performed using capillary electrophoresis-mass spectrometry (CE-MS) system (Agilent Technologies, Waldbronn, Germany) using the method described by Takahashi *et al.* [13] and Miyagi *et al.* [14], with minor modifications. Briefly, 50 mL of culture at an OD_730_ of 0.5 was harvested and the metabolites were extracted with ice-cold 50% (v/v) methanol containing internal standards (50 μM PIPES and 50 μM methionine sulfone). After filtration through a 3 kDa cutoff membrane (Amicon Ultra-0.5 3K, Millipore, Billerica, MA, USA), anionic metabolites (phosphorylated compounds and organic acids) were separated at −25 kV on a polyethylene glycol-coated capillary (100 cm × 50 µm i.d., DB-WAX, Agilent Technologies), using 20 mM ammonium acetate, pH 8.5, as a running buffer. For cationic metabolites (amino acids), an uncoated fused silica capillary (100 cm × 50 µm i.d., silica capillary tube, Agilent Technologies) was used at 25 kV with 1 M formic acid, pH 1.9, as a running buffer. To stabilize the MS analysis, 8 µL min**^−^**^1^ of sheath solution (5 mM ammonium acetate in 50% (v/v) methanol for anionic compounds or 0.1% formic acid (v/v) in 50% methanol for cationic compounds) was applied to the capillary using an isocratic HPLC pump (Agilent 1100 series) equipped with a 1:100 splitter. During the electrophoresis, quadrupole voltage (at ±3500 V) and the drying nitrogen gas flow (adjusted to 320 °C, 8 L·min**^−^**^1^) were maintained for 20–30 min. The quantitative accuracy of the analysis of various compounds was determined by measuring known concentrations of approximately 50 primary metabolites, using the Agilent ChemStation software (Rev.A.10.01). Multivariate analyses of the metabolome data (normalized with the Z score) were performed using the Statistical Package for the Social Sciences (SPSS v19.0, IBM, Armonk, NY, USA) and Excel 2010 (Microsoft, Redmond, WA, USA), as described by Miyagi *et al.* [15].

### 2.6. RNA Gel Blot Analysis

Isolation of total RNA and RNA gel blot analyses using a DIG RNA Labeling and Detection Kit (Roche) were performed as described previously [16].

To generate RNA probes by *in vitro* transcription, template DNA fragments were prepared by polymerase chain reaction (PCR) using the following primers: urtA-F (5'-ATGACTAACCCTTTTGGA-3') and SP6-urtA-R (5'-ATTTAGGTGACACTATAGAATACAGCAACCAATCCACCGCC-3'), PglnB-F (5'-AGAGGAAAAGTTTTTCGA-3') and T7-glnB-R (5'-TAATACGACTCACTATAGGGCGATTAAATAGCTTCGGTATC-3'), gnd-F (5'-GTCCACAACGGCATTGAG-3') and SP6-gnd-R (5'-ATTTAGGTGACACTATAGAATACTTTGCTTGAATTC-3'), sigE-F (5'-ATGAGCGATATGTCTTCC-3') and T7-sigE-R (5'-TAATACGACTCACTATAGGGCGAATCGCCCCTTCTTGGATC-3'), 0822-F (5'-AACATATGGCTAAATCAAACGCA-3') and 0822-R (5'-AAGGATCCTTACTCTTCTTCGTCGTC-3') and 0359-F (5'-AACATATGCCAAACGCCTCCACC-3') and 0359-R-Eco (5'-AAGAATTCTTATACTTCCTCTTCGTC-3'). The underlines indicate T7 polymerase recognition sequence (TAATACGACTCACTATAGGGCGA) or SP6 polymerase recognition sequence (ATTTAGGTGACACTATAGAATAC) added to the reverse primers in order to use PCR products directly as templates for *in vitro* transcription reaction. In the cases of *cyabrB2* (sll0822) and *cyabrB1* (sll0359), PCR products were cloned into the pT7Blue T-Vector containing a T7 polymerase recognition sequence, and the resultant plasmids were used as templates for *in vitro* transcription.

### 2.7. Immunoblot Analysis

Cell cultures at mid-logarithmic phase were harvested by centrifugation. After resuspension in breakage buffer (20 mM Tris-HCl, pH 8.0, 100 mM NaCl), cells were mixed with small amounts of glass beads (0.1 mm diameter; AsOne BZ-01) and disrupted using Pellet Pestle Motor (Kontes) twice for 1 min, followed each time by cooling on ice for 1 min. After addition of 2 x SDS sample buffer (62.5 mM Tris-HCl, pH 6.8, 4% SDS, 20% glycerol, 0.01% bromophenol blue), samples were centrifuged at 700 xg for 3 min to remove glass beads and cell debris. Aliquots of the supernatants containing 10 μg proteins per lane were loaded onto 15% polyacrylamide gel. After SDS gel electrophoresis and electroblotting onto PVDF membranes (Immobilon-P; Millipore), samples were probed with a rabbit polyclonal antibody raised against His-cyAbrB1 or His-cyAbrB2 recombinant protein. Goat anti-rabbit IgG conjugated to alkaline phosphatase was used as a secondary antibody.

## 3. Results

### 3.1. Decrease of Glycogen Content during Dark Incubation

**Figure 1:**
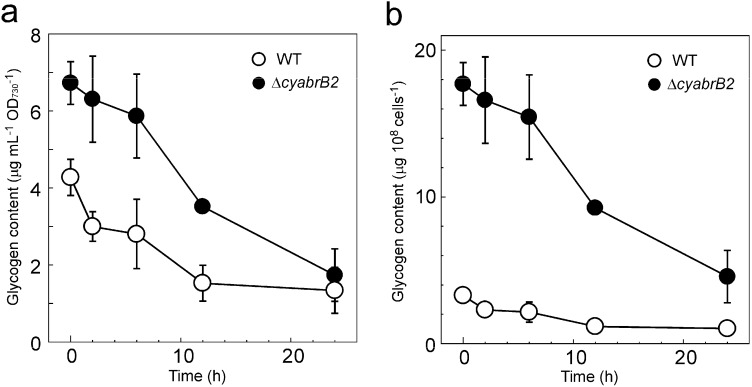
Decrease in glycogen content of the WT and the ∆*cyabrB2* mutant during dark incubation shown (**a**) based on OD_730_ and (**b**) on a per cell basis. At time 0, WT and the ∆*cyabrB2* mutant cultures grown under light conditions (20 μmol photons m^−2^ s^−1^, ambient CO_2_) were transferred to dark conditions. The mean and standard error values were calculated from the results of three independent experiments.

The change in glycogen content during dark incubation was measured in WT and the ∆*cyabrB2* mutant cells (Figure 1).

Under light conditions (time 0), the glycogen content of the WT and the ∆*cyabrB2* mutant was 4.3 ± 0.5 μg·mL*^−^*^1^·OD_730_*^−^*^1^ and 6.7 ± 0.6 μg·mL*^−^*^1^·OD_730_*^−^*^1^, respectively. The accumulation level in the mutant was lower than that observed by Kaniya *et al.* [10] (wild type 4.3 ± 1.6, ∆*cyabrB2* 14.6 ± 3.9 μg·mL*^−^*^1^·OD_730_*^−^*^1^). This difference may be caused by the difference in the growth stage of the culture used for the experiment. Considering that 1 mL of WT culture at OD_730_ = 1.0 contains 1.3 × 10^8^ cells, whereas the mutant culture of the same turbidity contains only 3.8 × 10^7^ cells [9], the glycogen contents on a per cell basis in WT and the mutant were 3.3 ± 0.4 μg·10^8^·cells*^−^*^1^ and 17.7 ± 1.5 μg·10^8^·cells*^−^*^1^, respectively. The glycogen content in both strains decreased linearly during dark incubation and the accumulation level on a per OD_730_ basis was almost halved within 12 h. This indicates that the ∆*cyabrB2* mutant is able to degrade accumulated glycogen granules when incubated under dark conditions.

**Figure 2:**
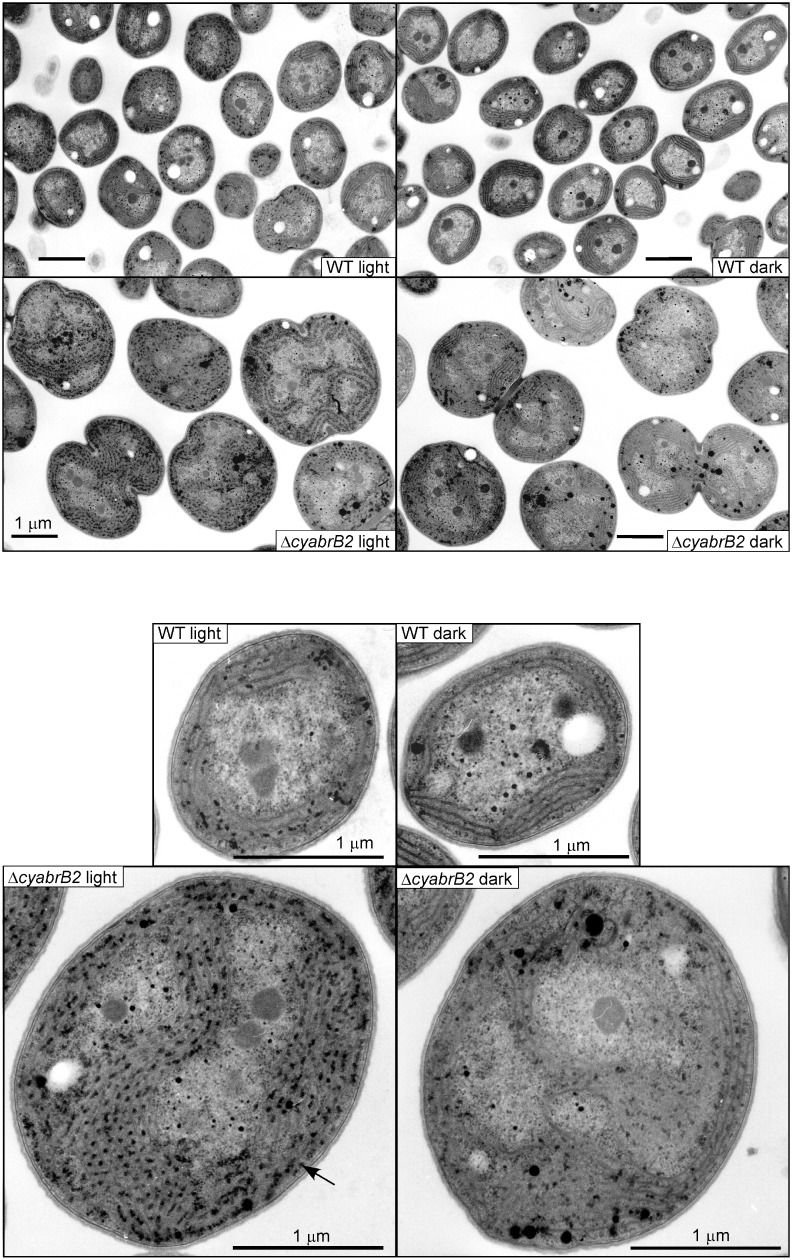
Electron micrographs of ultrathin sections of the WT and ∆*cyabrB2* mutant cells before and after incubation under dark conditions for 12 h. Magnified images of one cell are shown in the lower panels. The arrow indicates a glycogen granule. Bars = 1 μm.

Degradation of glycogen granules in the ∆*cyabrB2* mutant under dark conditions was confirmed by microscopic observation. Figure 2 shows electron micrographs of ultrathin sections of WT and ∆*cyabrB2* cells before and after incubation under dark conditions for 12 h.

The ∆*cyabrB2* mutant cells under light conditions were considerably larger than WT cells (diameter: WT 2.21 ± 0.18 μm, ∆*cyabrB2* mutant 3.82 ± 0.19 μm) [9] and accumulated substantial amounts of glycogen granules in the inter-thylakoid spaces, as previously reported [9,10]. For both strains, dark incubation had little effect on cell size or shape. The degree of glycogen granule accumulation was seen to decrease during dark incubation, although the extent of decrease varied somewhat among individual cells.

### 3.2. Changes in Respiratory Activity of the WT and the ∆cyabrB2 Mutant during Dark Incubation

Respiratory activity of the WT and the ∆*cyabrB2* mutant cells was measured as the rate of oxygen consumption in the dark using an oxygen electrode. The respiratory activity of both strains was not substantially affected during 12 h of dark incubation. The activity of the mutant was 55%–70% of that of WT based on OD_730_ (0 h: WT 0.075 ± 0.003, ∆*cyabrB2* 0.046 ± 0.004; 2 h: WT 0.069 ± 0.006, ∆*cyabrB2* 0.038 ± 0.003; 12 h: WT 0.066 ± 0.004, ∆*cyabrB2* 0.047 ± 0.005 μmol O_2_ consumed OD_730_*^−^*^1^·mL*^−^*^1^·h*^−^*^1^), and 2.4 times higher than that of WT on a per cell basis (0 h: WT 0.57 ± 0.02, ∆*cyabrB2* 1.21 ± 0.10; 2 h: WT 0.53 ± 0.05, ∆*cyabrB2* 1.01 ± 0.07; 12 h: WT 0.51 ± 0.03, ∆*cyabrB2* 1.24 ± 0.13 μmol O_2_ consumed 10^9^ cells*^−^*^1^·h*^−^*^1^). The accumulated glycogen appeared to undergo normal catabolism (Figure 1 and Figure 2) to maintain constant respiratory activity in the dark-incubated ∆*cyabrB2* mutant.

### 3.3. Changes in Transcript Levels in the WT and ∆cyabrB2 Mutant Cells under Dark Conditions

**Figure 3:**
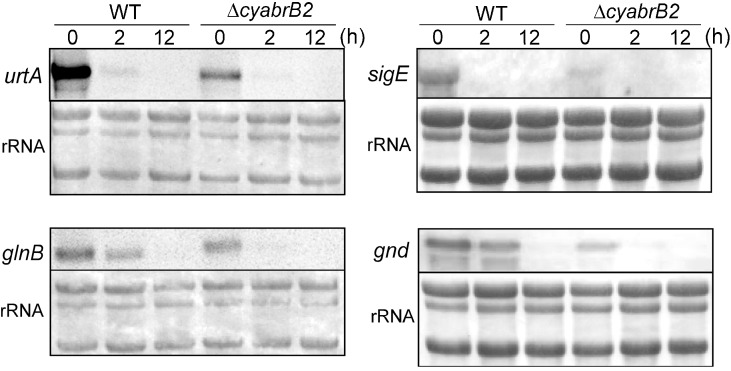
Changes in transcript levels of the WT and the ∆*cyabrB2* mutant cells upon shifting to dark conditions, as determined by RNA gel-blot analysis. The amount of total RNA loaded per lane was as follows: 2 μg for *urtA*, and *glnB*, 4 μg for *gnd*, and 6 μg for *sigE*. Total RNA was stained with methylene blue to compare loading within each blot.

Changes in the levels of various transcripts were compared between WT and the ∆*cyabrB2* mutant upon a shift to dark conditions (Figure 3). Expression levels of nitrogen-regulated genes, such as *urtA*, which encodes a subunit of the urea transporter, *glnB*, which encodes the nitrogen regulatory protein PII, and *sigE*, which encodes a group 2 sigma factor, as well as genes involved in sugar catabolism, such as *gnd*, which encodes 6-phosphogluconate dehydrogenase of the OPP, were all lower in the mutant than in WT under light conditions, as previously reported [5,10]. After the shift to dark conditions, the transcript levels of these genes markedly decreased within 2 h in both WT and the mutant, and were below the detection limit after 12 h.

### 3.4. Metabolome Analysis of the WT and ∆cyabrB2 Mutant Cells under Dark Conditions

**Figure 4:**
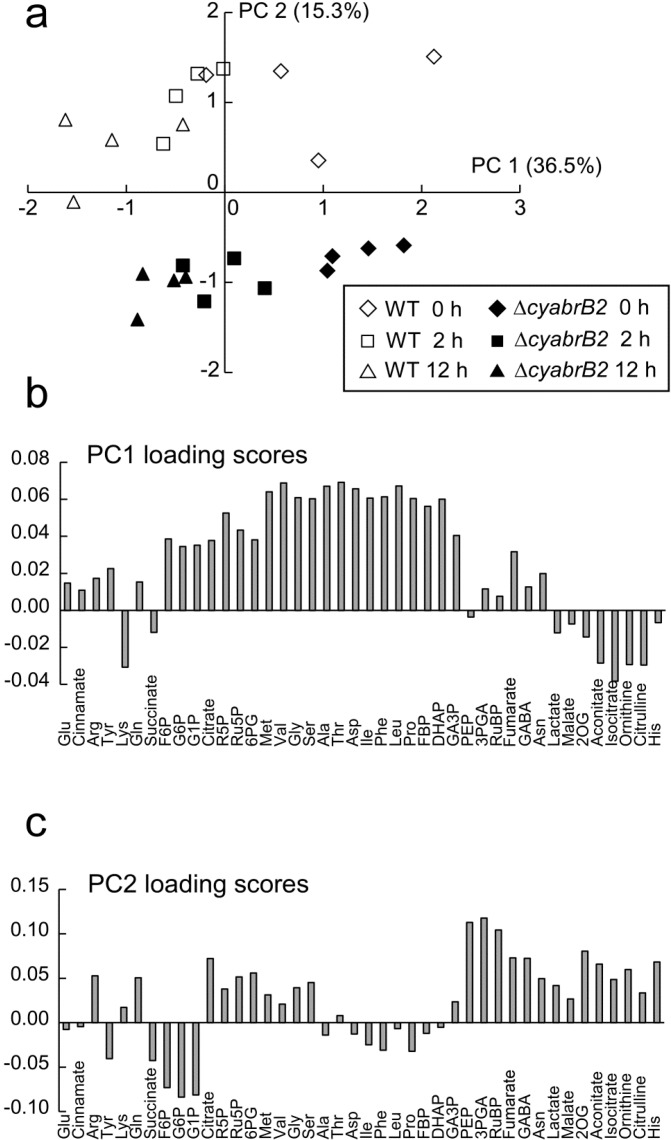
Principal component analysis of metabolites expressed as nmol g fresh weight^−1^ in WT and the ∆*cyabrB2* mutant before and after the shift to dark conditions. Scores of principal component analysis based on PC1 and PC2 components are presented in (**a**). Loadings scores of PC 1 (**b**) and PC 2 (**c**) components are also shown. The vertical axis shows each PC loading value.

We performed a metabolome analysis using CE-MS to investigate the consequences of the absence of cyAbrB2 expression on central carbon metabolism during dark incubation. Metabolite levels were evaluated in WT and ∆*cyabrB2* at 0, 2 and 12 h after the shift to dark conditions, and the results of a principal component analysis of more than 40 metabolites, including phosphorylated compounds, organic acids and amino acids are shown in Figure 4a.

Two principal components, PC1 and PC2, obtained from the metabolite data sets represented 51.8% of the total variance. The first component (PC1) reflected the duration of dark incubation. The time-dependent decrease of the PC1 value was observed both in WT and the ∆*cyabrB2* mutant. The loading scores of PC1 (Figure 4b) show that phosphorylated compounds and amino acids contributed to the positive direction, whereas most of the TCA metabolites contributed to the negative direction. In the second component (PC2), separation of WT (positive) and the mutant (negative) was observed (Figure 4a). Metabolites of the TCA, Calvin and ornithine cycles contributed to the positive direction, whereas sugar phosphates in the earlier steps of the glycolytic pathway contributed to the negative direction in the loading scores of PC2 (Figure 4c).

**Figure 5:**
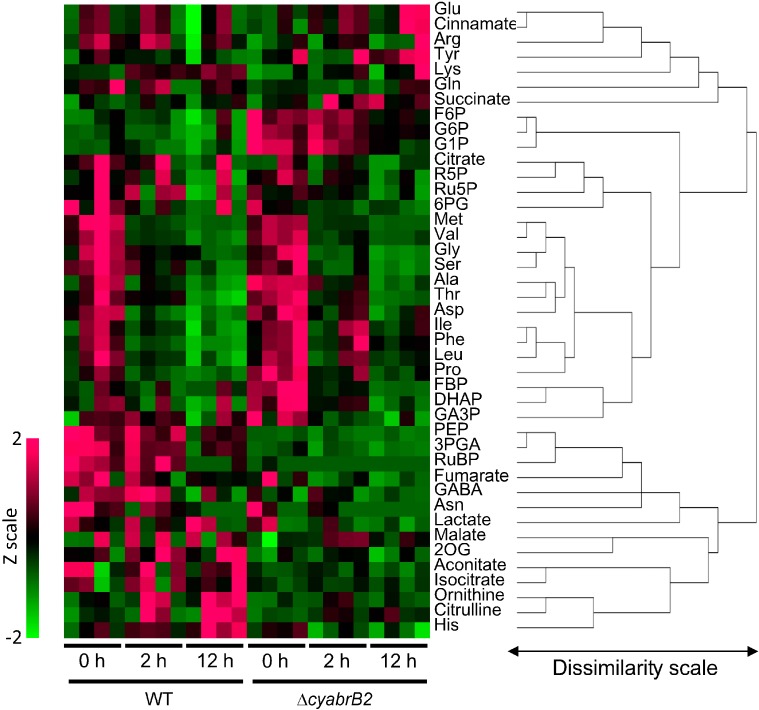
Hierarchical clustering and heat map of metabolites in the WT and ∆*cyabrB2* mutant after incubation under dark conditions (*n* = 4).

Hierarchical clustering analysis showed several distinctive differences in metabolite levels between strains and between light and dark conditions (Figure 5). To summarize: (1) The amounts of glucose-1-phosphate (G1P), glucose-6-phosphate (G6P) and fructose-6-phosphate (F6P) were significantly higher in the mutant at 0 h but decreased during dark incubation. Fructose 1,6-bisphosphate (FBP), dihydroxyacetone phosphate (DHAP) and glyceraldehyde-3-phosphate (GA3P) showed a similar trend; (2) Levels of some amino acids, such as methionine, valine, glycine, serine, alanine, threonine, aspartate, isoleucine, phenylalanine, leucine and proline, decreased during dark incubation, both in WT and the mutant; (3) Levels of 3-phosphoglycerate (3PGA), phosphoenolpyruvate (PEP) and ribulose 1,5-bisphosphate (RuBP) were significantly higher in WT at 0 h, but decreased during dark incubation; (4) The amounts of aconitate, isocitrate, 2OG, histidine, ornithine and citrulline increased during dark incubation in WT but not in the mutant.

**Figure 6:**
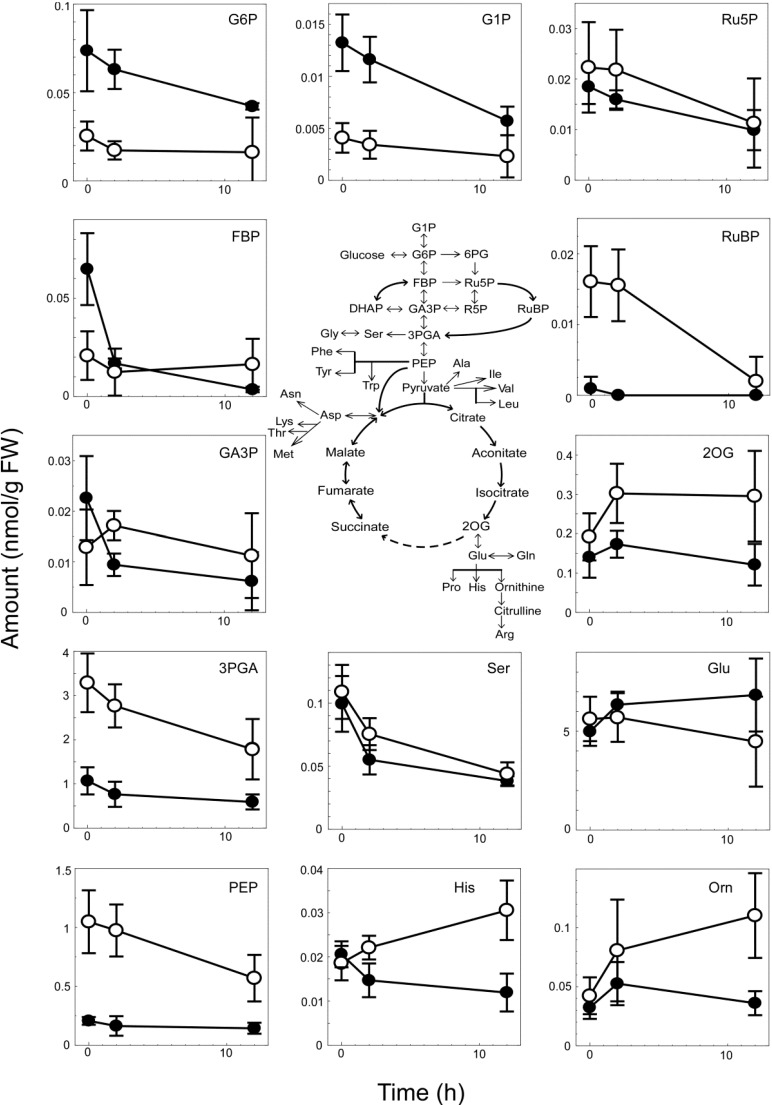
Changes in levels of major metabolites in the WT (open circles) and the ∆*cyabrB2* mutant (closed circles) during dark incubation for 0, 2 and 12 h. The mean and standard error values were calculated from the results of four independent experiments (nmol g fresh weight^−1^).

Figure 6 shows a quantitative representation of the major metabolites together with a map of central carbon metabolism.

Although significant differences in metabolite levels were observed between WT and ∆*cyabrB2* under light conditions, these differences tended to become smaller during dark incubation due to a general quantitative decrease in metabolite levels. However, notable exceptions were increases in 2OG, histidine, ornithine and citrulline in dark-incubated WT cells but not those of the mutant.

### 3.5. Light-Dependent Changes in Transcript and Protein Amounts of cyAbrBs

**Figure 7:**
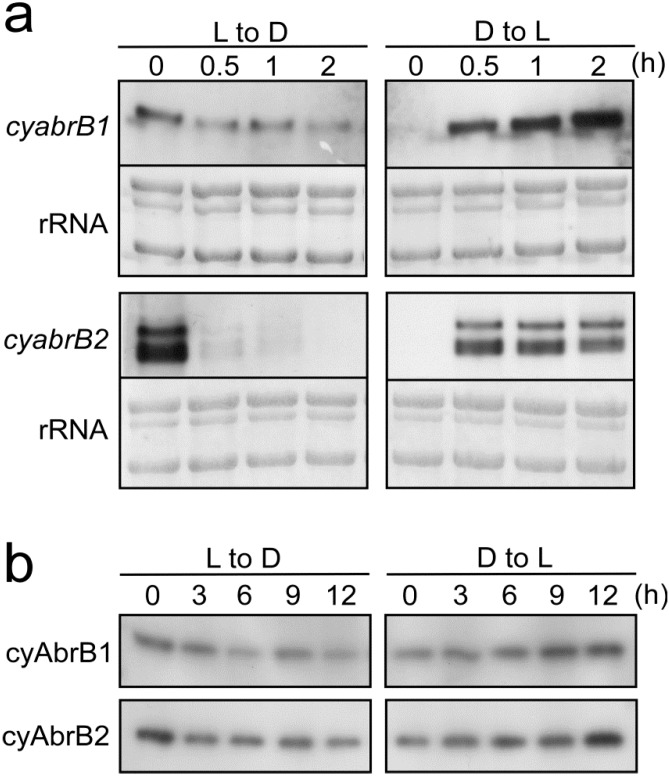
Changes in expression levels of cyAbrB1 and cyAbrB2 upon shifting from light to dark (L to D) and from dark to light (D to L) conditions. (**a**) *cyabrB1* and *cyabrB2* transcript levels, as determined by RNA gel blot analysis. 2 μg of total RNA was loaded per lane. Total RNA was stained with methylene blue to compare loading within each blot; (**b**) cyAbrB1 and cyAbrB2 protein levels as determined by immunoblot analysis. Anti His-cyAbrB1 or Anti His-cyAbrB2 antibodies were used to detect cyAbrB proteins in crude cell extracts. 10 μg proteins per lane were loaded onto a 15% polyacrylamide gel.

Metabolome analysis suggested that the contribution of cyAbrB2 to metabolic regulation upon shifting to dark conditions is minimal, at least in terms of central carbon metabolism. The question therefore arises, are expression levels of cyAbrB2 affected by light availability? Figure 7a shows the changes in transcript levels of *cyabrB1* and *cyabrB2* upon a shift from light to dark, and dark to light.

Interestingly, levels of the *cyabrB2* transcripts rapidly decreased below the detection limit within 30 min of dark incubation (l to d). Conversely, when cultures were re-illuminated after 6 h in the dark, a marked accumulation of *cyabrB2* transcripts was observed within 30 min (d to l). *cyabrB1* transcript levels showed similar light response, but differed from *cyabrB2* in that low amounts of transcripts were still detected after 2 h of dark incubation. Immunoblot analysis, using antisera that specifically recognize cyAbrB1 or cyAbrB2 (Figure 7b), showed that the levels of both proteins also decreased following dark incubation and increased as a result of re-illumination.

### 3.6. Growth Property and Glycogen Content of the WT and ∆cyabrB2 Mutant Cells during Diurnal Light-Dark Cycles

**Figure 8:**
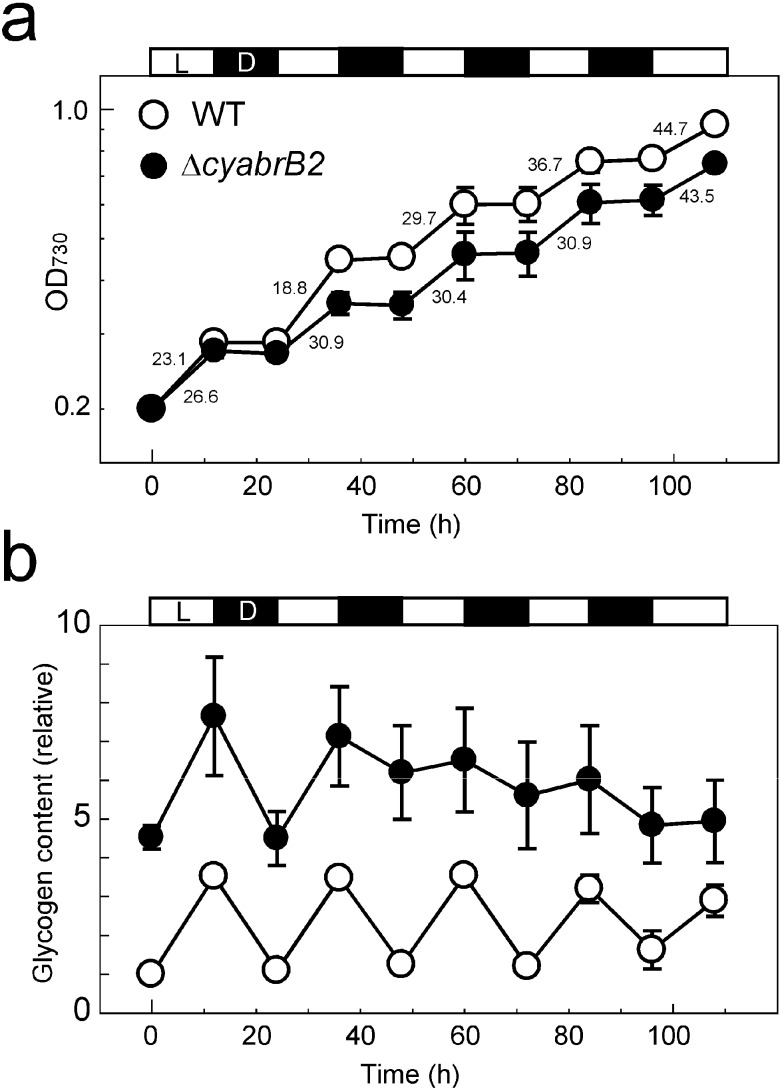
Growth and changes in glycogen content of the WT and ∆*cyabrB2* mutant cultures under 12 h:12 h light (20 μmol photons m^−2^s^−1^) -dark cycle. (**a**) Growth of the culture based on OD_730_. The values shown within the figure are the doubling times (h) at each light period; (**b**) Glycogen content based on OD_730_, shown as fold changes compared to the level in WT at time 0. The mean and standard error values were calculated from the results of three independent experiments.

To assess the impact of the absence of cyAbrB2 on cell growth and cellular metabolism during diurnal light-dark cycle, we monitored growth and the cellular glycogen content of WT and the ∆*cyabrB2* mutant cells cultured under 12 h:12 h light-dark cycle conditions (Figure 8).

When grown under continuous light conditions of 20 μmol photons m*^−^*^2^ s*^−^*^1^, the doubling time of WT and mutant cells was 18 h and 26 h, respectively. A similar growth pattern was observed at the first and the second light periods of light-dark circle (Figure 8a). However, after the third light period, the doubling time of both strains similarly increased to 30-45 h. At time 0, the glycogen content of the WT and the ∆*cyabrB2* mutant was 3.2 ± 0.5 μg mL*^−^*^1^·OD_730_*^−^*^1^ and 16.0 ± 1.3 μg·mL*^−^*^1^·OD_730_*^−^*^1^, respectively. The glycogen content, measured at the end of each light or dark period, exhibited a diurnal oscillatory pattern in WT cells (Figure 8b). However, in the ∆*cyabrB2* mutant, while the glycogen content showed a substantial decrease in the first dark period, the rate of decrease was significantly lower in the second dark period and subsequently the regular marked oscillatory pattern was not apparent. This suggests that cellular metabolism of the mutant was disordered when grown under light-dark cycle conditions, although growth rate of the culture was not affected. Thus, growth rate and glycogen utilization appear to not be coupled, since a short light/dark cultivation time has a major effect on growth but not on glycogen cycling, while longer cultivation under such conditions results in equivalent growth rate but difference in glycogen utilization.

## 4. Discussion

### 4.1. Physiological Roles of cyAbrB2 under Dark Conditions

In this study, we performed a metabolome analysis to elucidate the role of cyAbrB2 in metabolic regulation under dark conditions. The results indicate that cyAbrB2 makes less significant contribution to metabolite levels during dark incubation, although the metabolic phenotypes of the ∆*cyabrB2* mutant under light conditions were more notable in this study than in our previous report [10]. In the previous study, which examined the effects of changes in trophic conditions, the levels of sugar phosphates were 1.5–2 times higher and the amounts of pyruvate and 2OG were lower in the mutant than in the WT under photoautotrophic conditions. A decrease in RuBP content in the mutant was observed but was not substantial (66% of WT level). In this current study, the levels of G1P, G6P and FBP in the mutant were approximately three times higher than those in WT (Figure 6). Although the amounts of the OPP metabolites, such as 6PG, Ru5P and R5P, were similar between strains, the levels of the Calvin cycle metabolites RuBP and 3PGA were significantly lower in the mutant. In contrast to the enhanced accumulation of G1P, G6P and FBP, the amounts of the metabolites in the later stages of the glycolytic chain, 3PGA and PEP, were low in the mutant. Although pyruvate was not detected in this study, its concentration may also be lower in the mutant, according to the observations of Kaniya *et al.* [10]. The data obtained in this study, together with the results of Kaniya *et al.* [10], suggest that the decreased activity of the downstream metabolic pathways, and especially the later steps in the glycolytic pathway, results in accumulation of glycogen within the ∆*cyabrB2* mutant cells under light conditions.

Upon a shift to dark conditions, the differences in metabolite levels between WT and the ∆*cyabrB2* mutant became less conspicuous, due to a general decrease in metabolite levels. A significant decrease in glycogen and sugar phosphates levels in the mutant (Figure 1, Figure 2 and Figure 6) indicates that a defect in sugar catabolism that was observed under light conditions was relieved in the dark-incubated mutant. Respiratory activity, measured as O_2_ consumption rate, was not substantially affected by light availability in either of the strains, and the activity of the mutant was 55%–70% of that of WT, based on OD_730_, and 2.4 times higher than that of WT on a per cell basis. In both strains, the dark-respiratory activity is likely maintained by the supply of NADPH from the OPP pathway, through which glucose residues derived from glycogen are oxidized.

In both strains, a decrease in the abundance of amino acids derived from 3PGA, pyruvate and oxaloacetate was observed in dark conditions. These amino acids may be converted to intermediates of central carbon metabolism to support energy production in dark. However, metabolites derived from 2OG showed a contrasting pattern: glutamate, the major amino group donor in amino acid biosynthesis, was abundant in both strains irrespective of light conditions, whereas 2OG, histidine, ornithine and citrulline levels increased only in the WT under dark conditions (Figure 5 and Figure 6). We have previously reported that amounts of these metabolites in the light-grown ∆*cyabrB2* mutant are lower than those in WT probably due to insufficient nitrogen assimilation [10]. The lack of accumulation of these metabolites in the dark-grown mutant may also be related to the defect in nitrogen assimilation.

The data related to glycogen content shown in Figure 8b may represent an important clue for elucidating the physiological role of cyAbrB2 under diurnal light-dark conditions. Cultivation of the ∆*cyabrB2* mutant under a 12-h light-dark diurnal cycle revealed the defect in glycogen metabolism after the second dark period. Absence of cyAbrB2 may cause the inefficient activation of metabolism required for growth under light conditions. Further metabolome analysis at the transition from the second to the third light-dark cycle will help clarify the physiological role of cyAbrB2 under diurnal light-dark conditions.

### 4.2. Regulatory Mechanisms Working under Dark Conditions

Under light conditions, cyAbrB2 acts as an important metabolic regulator by activation of genes such as *glnB*, which encodes a PII protein that functions as both a sensor and a regulator of carbon and nitrogen balance [17], *sigE* encoding a group two sigma factor involved in the positive regulation of sugar catabolism [18] and *gnd* encoding 6PGDH, one of the key enzymes of the OPP pathway [10]. Upon the transition from light to dark, transcript levels of these genes markedly decreased within 2 h in both WT and the ∆*cyabrB2* mutant (Figure 3), indicating that positive regulation by cyAbrB2 ceases under dark conditions. Instead, different systems of metabolic regulation must be active under dark conditions. One possible candidate is circadian control. Kucho *et al.* [19] reported that several rate-limiting enzymes in the sugar catabolic pathways, such as phosphofructokinase (sll1196), G6PDH (slr1843), 6PGDH (sll0329), pyruvate kinase (sll1275) and glucokinase (sll0593), were under the control of circadian regulation in *Synechocystis* sp. PCC 6803. Furthermore, several regulatory factors have been suggested to operate in the circadian regulation of metabolic processes. Singh and Sherman [20] reported that the histidine kinase Hik8 (Sll0750) positively regulates genes related to glycolysis and the OPP pathway. The deletion mutant of *hik8* accumulated glycogen granules but was unable to catabolize them for heterotrophic growth. Hik8 may be indeed be involved in circadian regulation since SasA, the Hik8 homolog in *Synechococcus* sp. PCC 7942, was shown to be associated with KaiABC, the central oscillator of circadian rhythm [21]. Recently, the response regulator Sll1330 has been suggested to be located downstream of Hik8 in the signal transduction pathway [22]. Sll1330 is involved in the activation of glycolytic genes under light-activated heterotrophic growth (LAHG) conditions [23]. The facts that sll1330 was listed as a circadian-regulated gene [19] and induction of sll1330 under LAHG conditions was not observed in the *hik8*–deleted mutant [22] suggests that Sll1330 is located downstream of Hik8 and plays a role in the circadian regulation of glycolytic genes. Kucho *et al.* [19] reported that SigE was also circadian-regulated and the expression of *sigE* peaked just prior to the maximal expression of sugar catabolic genes.

Although the transcript and protein levels of cyAbrB1 and cyAbrB2 changed significantly upon the shift from light to dark, or *vice versa* (Figure 7), they were not listed as cycling genes in Kucho *et al.* [19]. We suggest that cyAbrB2 may have a role in the activation and coordination of cellular metabolism independent of circadian regulation. Its expression may be redox-regulated at transcriptional and/or post-transcriptional levels, and it has been reported that glutathionylation of the well-conserved cysteine residue of cyAbrB2 affects its DNA binding activity [24]. Further investigation of the regulatory mechanism of cyAbrB2 activity will be important for gaining a better understanding of metabolic regulation in cyanobacteria.

## 5. Conclusions

In this study, we examined the role of cyAbrB2 in cellular metabolic regulation under dark conditions by characterizing WT and the *cyabrB2*-disrupted mutant cells, using a metabolomic approach. Glycogen and metabolites in the earlier steps of glycolysis accumulated to high levels in the ∆*cyabrB2* mutant under light conditions, whereas they were actively catabolized under dark conditions. We also determined that levels of cyAbrB1 and cyAbrB2 transcripts and proteins were highly responsive to light availability, and that cyAbrB2 is essential for maintaining the diurnal oscillatory pattern of glycogen under a 12-h light-dark diurnal cycle. We draw two conclusions concerning the function of cyAbrB2 and the pathways it regulates. The first is that cyAbrB2 is responsible for the activation of sugar catabolism specifically during the daytime. The second is that the reallocation of accumulated glycogen to synthetic pathways that result in useful targeted products may be possible during the night when sugar catabolism can be activated in the ∆*cyabrB2* mutant. The success of such a metabolic engineering strategy will likely depend on an improved understanding of how carbon is allocated between dark respiration related metabolism and the synthesis of specific biotechnology related products.
